# Supplementary material for: Neutralizing monoclonal antibodies against the Gc fusion loop region of Crimean–Congo hemorrhagic fever virus
Source: PLoS Pathog. 2024 Feb 1;20(2):e1011948. doi: 10.1371/journal.ppat.1011948 (PMC10863865; doi:10.1371/journal.ppat.1011948)
Supplement: S1 Fig — (PDF) [file ppat.1011948.s001.pdf]

S1 Fig.

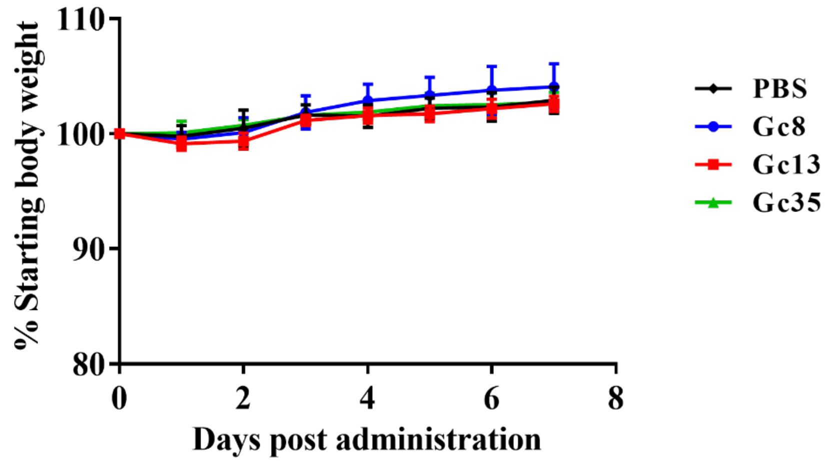

S1 Fig. Body weight curve of C57BL/6J-IFNAR-/- mice (5 mice per group) administered with Gc8, Gc13 and Gc35 at a dose of 50 mg/kg, and the same volume of PBS control, via the intraperitoneal route
