# Supplementary material for: Neutralizing monoclonal antibodies against the Gc fusion loop region of Crimean–Congo hemorrhagic fever virus
Source: PLoS Pathog. 2024 Feb 1;20(2):e1011948. doi: 10.1371/journal.ppat.1011948 (PMC10863865; doi:10.1371/journal.ppat.1011948)
Supplement: S2 Fig — Representative negative staining micrographs of CCHFV Gc (A), the Gc-Gc8 complex (B), and the Gc-Gc13 complex (C). Scale bar: 50 nm. (PDF) [file ppat.1011948.s002.pdf]

S2 Fig.

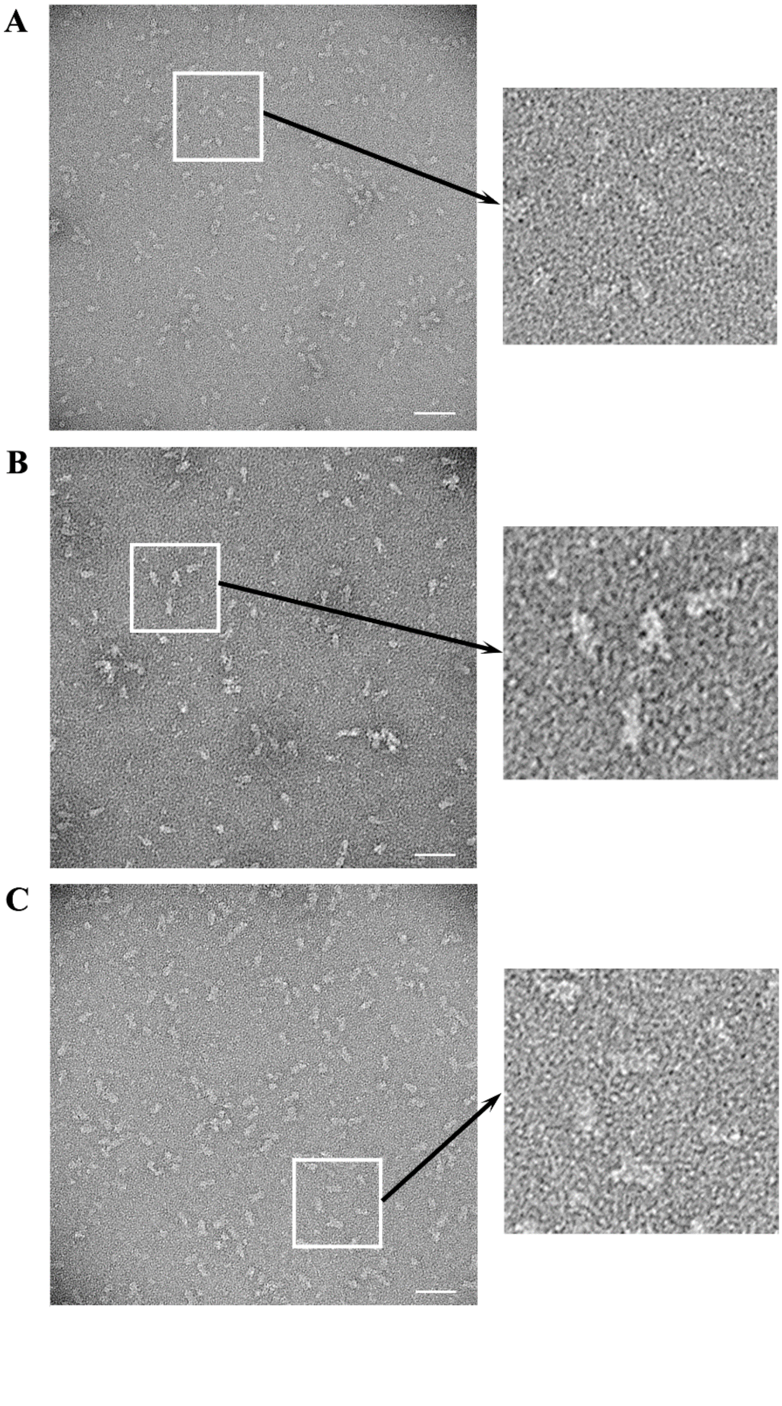

S2 Fig. Representative negative staining micrographs of CCHFV Gc (A), the Gc-Gc8 complex (B), and the Gc-Gc13 complex (C). Scale bar: 50 nm.
