# Supplementary material for: Neutralizing monoclonal antibodies against the Gc fusion loop region of Crimean–Congo hemorrhagic fever virus
Source: PLoS Pathog. 2024 Feb 1;20(2):e1011948. doi: 10.1371/journal.ppat.1011948 (PMC10863865; doi:10.1371/journal.ppat.1011948)
Supplement: S4 Fig — Cryo-EM data processing of Gc-Gc13 complex: representative micrograph (A), 2D class averages (B), 3D classes (C), and local refinement of Gc-Gc13 interface(D). (PDF) [file ppat.1011948.s004.pdf]

S4 Fig.

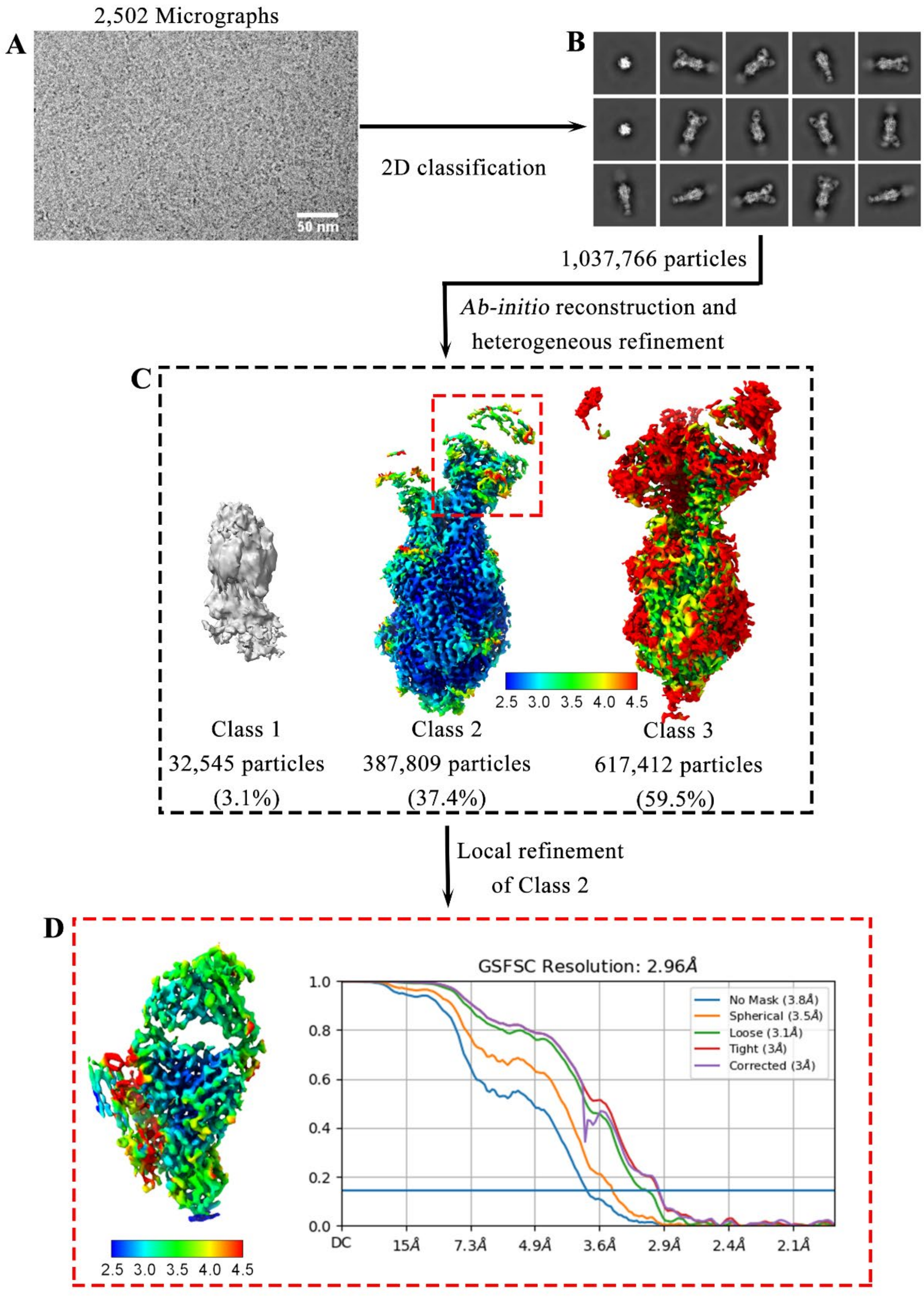

S4 Fig. Cryo-EM data processing of Gc-Gc13 complex: representative micrograph (A), 2D class averages (B), 3D classes (C), and local refinement of Gc-Gc13 interface(D).
