# Supplementary material for: Neutralizing monoclonal antibodies against the Gc fusion loop region of Crimean–Congo hemorrhagic fever virus
Source: PLoS Pathog. 2024 Feb 1;20(2):e1011948. doi: 10.1371/journal.ppat.1011948 (PMC10863865; doi:10.1371/journal.ppat.1011948)
Supplement: S5 Fig — Phylogenetic tree was analyzed using MEGA version 6.0, distances were calculated by Kimura’s 2-parameter and a phylogenetic tree was plotted by the neighbor-joining method based on full segment of M. Thirty-three isolates were classified into eight clades. (PDF) [file ppat.1011948.s005.pdf]

S5 Fig.

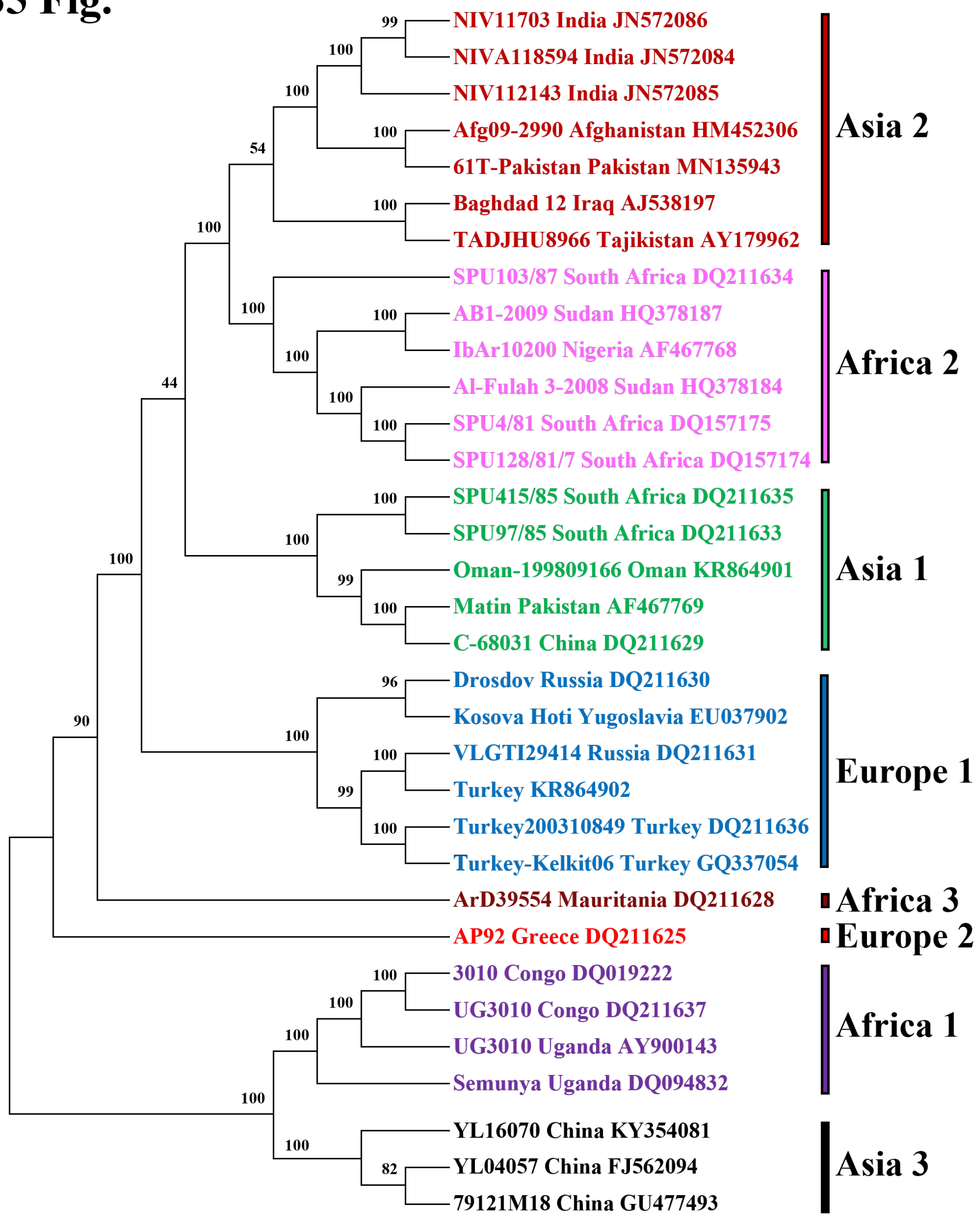

S5 Fig. CCHFV M segment based phylogenetic analysis. Phylogenetic tree was analyzed using MEGA version 6.0, distances were calculated by Kimura's 2-parameter and a phylogenetic tree was plotted by the neighbor-joining method based on full segment of M. Thirty-three isolates were classified into eight clades.
