# Supplementary material for: Neutralizing monoclonal antibodies against the Gc fusion loop region of Crimean–Congo hemorrhagic fever virus
Source: PLoS Pathog. 2024 Feb 1;20(2):e1011948. doi: 10.1371/journal.ppat.1011948 (PMC10863865; doi:10.1371/journal.ppat.1011948)
Supplement: S1 Table — (PDF) [file ppat.1011948.s006.pdf]

S1 Table. Cryo-EM data collection and refinement statistics.

	CCHFV Gc-Trimer with Gc13 Fab	CCHFV Gc-Gc8 Fab (Local refinement)	CCHFV Gc-Gc13 Fab (Local refinement)
PDB code	8JKD	8JLW	8JLX
EMDB code	EMD-36368	EMD-36406	EMD-36407
**Data collection and processing**			
Microscope	JEOL CRYO ARM 300	JEOL CRYO ARM 300	JEOL CRYO ARM 300
Voltage (kV)	300	300	300
Magnification	50,000	50,000	50,000
Pixel size (Å/pix)	0.95	0.95	0.95
Exposure (e^{-}/Å^2)	40	40	40
Defocus range (μm)	0.5-2.5	0.5-2.5	0.5-2.5
Final particle images (no.)	465,995	591,671	387,809
Symmetry imposed	C1	C1	C1
Map resolution (Å)	2.6	3.1	3
FSC threshold	0.143	0.143	0.143
Map sharpening B factor (Å^2)	-98.5	-108.5	-104.2
**Model refinement**			
Model composition			
Chains	3	3	3
Non-hydrogen atoms	10,960	2,157	2,277
Protein residues	1403	280	293
B factors for protein (Å^2)	29.76	35.71	34.46
R.m.s. deviations			
Bond length (Å)	0.003(0)	0.005(0)	0.004(0)
Bond angles (º)	0.632(0)	0.694(0)	0.746(0)
Validation			
Poor rotamers (%)	0	0	0
Clash score	7.32	11.03	12.5
MolProbity score	1.84	2.09	2.21
Ramachandran plot			
Favored (%)	93.35	90.74	88.07
Allowed (%)	6.65	9.26	11.93
Outliers (%)	0	0	0
