# Supplementary material for: Neutralizing monoclonal antibodies against the Gc fusion loop region of Crimean–Congo hemorrhagic fever virus
Source: PLoS Pathog. 2024 Feb 1;20(2):e1011948. doi: 10.1371/journal.ppat.1011948 (PMC10863865; doi:10.1371/journal.ppat.1011948)
Supplement: S2 Table — (PDF) [file ppat.1011948.s007.pdf]

S2 Table. Interactions between Gc8 light chain variable (VL) region and Gc.

Interaction Loop[a]	Chain: Residue	Hydrogen Bond	Buried Surface Area, Å^2
CDRL1			40.21
	L:TYR 30		0.46
	L:PHE 32		39.75
CDRL3			153.56
	L:HIS 91		23.69
	L:TYR 92	H	45.14
	L:GLY 93		13.61
	L:ILE 94		50.36
	L:LEU 96		20.76
"*cd*" loop			212.7
	A:THR1196	H	54.83
	A:TRP1197		86.7
	A:TRP1199		71.17

[a] For simplicity, residues neighboring the loop regions are also included. The "*bc*" loop contains residues Thr1164–Cys1169; the "*cd*" loop contains His1187–Gly1204; the "*ij*" loop contains Cys1360–Pro1366.
