# Supplementary material for: Neutralizing monoclonal antibodies against the Gc fusion loop region of Crimean–Congo hemorrhagic fever virus
Source: PLoS Pathog. 2024 Feb 1;20(2):e1011948. doi: 10.1371/journal.ppat.1011948 (PMC10863865; doi:10.1371/journal.ppat.1011948)
Supplement: S3 Table — (PDF) [file ppat.1011948.s008.pdf]

S3 Table. Interactions between Gc8 heavy chain variable (VH) region and Gc.

Interaction Loop	Chain: Residue	Hydrogen Bond	Buried Surface Area, Å^2
CDRH1			32.37
	H:SER 33		17.92
	H:HIS 35		14.45
CDRH2			192.92
	H:TRP 47		12
	H:ALA 50		9.26
	H:ILE 51		1.61
	H:TYR 52		60.97
	H:ASN 55		21.92
	H:ASP 57		55.99
	H:SER 59		31.17
CDRH3			222.36
	H:ASP 100		3.68
	H:GLN 101		12.61
	H:TYR 102	H	125.01
	H:TYR 103		59.57
	H:GLY 104		21.49
"*bc*" loop			79.77
	A:CYS1165	H	17.57
	A:THR1166		62.2
"*cd*" loop			406.82
	A:ASN1190		13.03
	A:TRP1191		129.23
	A:ARG1192		19.48
	A:TRP1197		24.09
	A:CYS1198		2.01
	A:TRP1199		106.19
	A:GLY1200		26.22
	A:VAL1201		56.99
	A:THR1203		29.58
