# Supplementary material for: Neutralizing monoclonal antibodies against the Gc fusion loop region of Crimean–Congo hemorrhagic fever virus
Source: PLoS Pathog. 2024 Feb 1;20(2):e1011948. doi: 10.1371/journal.ppat.1011948 (PMC10863865; doi:10.1371/journal.ppat.1011948)
Supplement: S4 Table — (PDF) [file ppat.1011948.s009.pdf]

S4 Table. Interactions between Gc13 light chain variable (VL) region and Gc.

Interaction Loop	Chain: Residue	Hydrogen Bond	Buried Surface Area, Å²
CDRL1			73.13
	L:SER 30		1.23
	L:TYR 32	H	71.9
CDRL2			54.11
	L:TYR 50	H	50.77
	L:ARG 53		3.34
CDRL3			71.04
	L:GLY 91		10.42
	L:SER 92		15.93
	L:LEU 94		7.51
	L:TRP 96		37.18
"*cd*" loop			190.34
	A:ASN1190		4.01
	A:TRP1191	H	77.2
	A:TRP1199		36.45
	A:VAL1201		64.96
	A:THR1203		7.72
