# Supplementary material for: Neutralizing monoclonal antibodies against the Gc fusion loop region of Crimean–Congo hemorrhagic fever virus
Source: PLoS Pathog. 2024 Feb 1;20(2):e1011948. doi: 10.1371/journal.ppat.1011948 (PMC10863865; doi:10.1371/journal.ppat.1011948)
Supplement: S5 Table — (PDF) [file ppat.1011948.s010.pdf]

S5 Table. Interactions between Gc13 heavy chain variable (VH) region and Gc.

Interaction Loop	Chain: Residue	Hydrogen Bond	Buried Surface Area, Å²
CDRH1			130.52
	H:PHE 29		41.27
	H:ASP 31	H	40.3
	H:TYR 32		11.36
	H:ALA 33		23.47
	H:HIS 35		14.12
CDRH2			200.61
	H:TRP 47		2.97
	H:VAL 50		23.09
	H:SER 52		19.65
	H:TYR 54	H	64
	H:ASN 55		1.75
	H:GLU 57		44.17
	H:THR 58		14.32
	H:ASP 59	H	30.66
CDRH3			256.48
	H:GLY 99		15.05
	H:TYR 100		20.78
	H:TYR 101	H	139.1
	H:GLN 102	H	81.55
"*bc*" loop			61.02
	A:ALA1163	H	12.76
	A:THR1164		7.37
	A:CYS1165		12.68
	A:THR1166		27.92
	A:GLY1167		0.29
"*cd*" loop			475.41
	A:TRP1191		38.23
	A:ASN1194	H	8.01
	A:PRO1195		7.82
	A:THR1196	H	101.84
	A:TRP1197	H	146.26
	A:CYS1198		12.19
	A:TRP1199	H	139.77
	A:GLY1200	H	1.84
	A:VAL1201		19.08
	A:THR1203		0.37
"*ij*" loop			43.62
	A:MET1362		0.12
	A:GLY1363	H	11.61
	A:TRP1365		31.89
