# Supplementary material for: Neutralizing monoclonal antibodies against the Gc fusion loop region of Crimean–Congo hemorrhagic fever virus
Source: PLoS Pathog. 2024 Feb 1;20(2):e1011948. doi: 10.1371/journal.ppat.1011948 (PMC10863865; doi:10.1371/journal.ppat.1011948)
Supplement: S6 Table — (PDF) [file ppat.1011948.s011.pdf]

S6 Table. The residues/atoms involved in hydrogen bonds between Fab and the fusion loops.

Residues from Fab	Residues from fusion loops	Distance (Å)
**Gc8 Heavy chain**		
TYR102[O]	CYS1165[SG] ("*bc*" loop)	3.66
**Gc8 Light chain**		
TYR92[O]	THR1196[OG1] ("*cd*" loop)	3.25
**Gc13 Heavy chain**		
TYR54[OH]	ALA1163[O] ("*bc*" loop)	3.67
GLN102[NE2]	ASN1194[OD1] ("*cd*" loop)	3.73
TYR101[N]	THR1196[O] ("*cd*" loop)	3.80
ASP31[OD2]	TRP1197[NE1] ("*cd*" loop)	2.30
ASP59[OD2]	TRP1199[NE1] ("*cd*" loop)	3.88
GLN102[NE2]	GLY1200[O] ("*cd*" loop)	2.61
TYR101[OH]	GLY1363[O] ("*ij*" loop)	3.85
**Gc13 Light chain**		
TYR32[OH]	TRP1191[N] ("*cd*" loop)	3.47
TYR50[OH]	TRP1191[NE1] ("*cd*" loop)	2.69
