# Supplementary material for: Neutralizing monoclonal antibodies against the Gc fusion loop region of Crimean–Congo hemorrhagic fever virus
Source: PLoS Pathog. 2024 Feb 1;20(2):e1011948. doi: 10.1371/journal.ppat.1011948 (PMC10863865; doi:10.1371/journal.ppat.1011948)
Supplement: S7 Table — (PDF) [file ppat.1011948.s012.pdf]

Table S7. Fusion loop sequences of different CCHFV isolates.

Clades	CCHFV isolates[a]	*bc* loop[b]	*cd* loop	*ij* loop
Asia 2	NIV11703 India JN572086	TCTGDC	HSRNWRCNPTWCWGVGTG	CNMGDWP
	NIVA118594 India JN572084	TCTGDC	HSRNWRCNPTWCWGVGTG	CNMGDWP
	NIV112143 India JN572085	TCTGDC	HSRNWRCNPTWCWGVGTG	CNMGDWP
	Afg09-2990 Afghanistan HM452306	TCTGDC	HSRNWRCNPTWCWGVGTG	CNMGDWP
	61T-Pakistan Pakistan MN135943	TCTGDC	HSRNWRCNPTWCWGVGTG	CNMGDWP
	Baghdad 12 Iraq AJ538197	TCTGDC	HSRNWRCNPTWCWGVGTG	CNMGDWP
	TADJHU8966 Tajikistan AY179962	TCTGDC	HSRNWRCNPTWCWGVGTG	CNMGDWP
Africa 2	SPU103/87 South Africa DQ211634	TCTGDC	HSRNWRCNPTWCWGVGTG	CNMGDWP
	AB1-2009 Sudan HQ378187	TCTGDC	HSRNWRCNPTWCWGVGTG	CNMGDWP
	IbAr10200 Nigeria AF467768	TCTGDC	HSRNWRCNPTWCWGVGTG	CNMGDWP
	Al-Fulah 3-2008 Sudan HQ378184	TCTGDC	HSRNWRCNPTWCWGVGTG	CNMGDWP
	SPU4/81 South Africa DQ157175	TCTGDC	HSRNWRCNPTWCWGVGTG	CNMGDWP
	SPU128/81/7 South Africa DQ157174	TCTGDC	HSRNWRCNPTWCWGVGTG	CNMGDWP
Asia 1	SPU415/85 South Africa DQ211635	TCTGDC	HSRNWRCNPTWCWGVGTG	CNMGDWP
	SPU97/85 South Africa DQ211633	TCTGDC	HSRNWRCNPTWCWGVGTG	CNMGDWP
	Oman-199809166 Oman KR864901	TCTGDC	HSRNWRCNPTWCWGVGTG	CNMGDWP
	Matin Pakistan AF467769	TCTGDC	HSRNWRCNPTWCWGVGTG	CNMGDWP
	C-68031 China DQ211629	TCTGDC	HSRNWRCNPTWCWGVGTG	CNMGDWP
Europe 1	Drosdov Russia DQ211630	TCTGDC	HSRNWRCNPTWCWGVGTG	CNMG**N**WP
	Kosova Hoti Yugoslavia EU037902	TCTGDC	HSRNWRCNPTWCWGVGTG	CNMGDWP
	VLGTI29414 Russia DQ211631	TCTGDC	HSRNWRCNPTWCW**E**VGTG	CNMGDWP
	Turkey KR864902	TCTGDC	HSRNWRCNPTWCWGVGTG	CNMGDWP
	Turkey200310849 Turkey DQ211636	TCTGDC	HSRNWRCNPTWCWGVGTG	CNMGDWP
	Turkey-Kelkit06 Turkey GQ337054	TCTGDC	HSRNWRCNPTWCWGVGTG	CNMGDWP
Africa 3	ArD39554 Mauritania DQ211628	TCTGDC	HSRNWRCNPTWCWGVGTG	CNMGDWP
Europe 2	AP92 Greece DQ211625	TCTG**N**C	HSRNWRCNPTWCWGVGTG	CNMGDWP
Africa 1	3010 Congo DQ019222	TCTGDC	HSRNWRCNPTWCWGVGTG	CNMGDWP
	UG3010 Congo DQ211637	TCTGDC	HSRNWRCNPTWCWGVGTG	CNMGDWP
	UG3010 Uganda AY900143	TCTGDC	HSRNWRCNPTWCWGVGTG	CNMGDWP
	Semunya Uganda DQ094832	TCTGDC	HSRNWRCNPTWCWGVGTG	CNMGDWP
Asia 3	YL16070 China KY354081	TCTGDC	HSRNWRCNPTWCWGVGTG	CNMGDWP
	YL04057 China FJ562094	TCTGDC	HSRNWRC**S**PTWCWGVGTG	CNMGDWP
	79121M18 China GU477493	TCTGDC	HSRNWRCNPTWCWGVGTG	CNMGDWP

[a]The color of isolates names are same with **Fig. S5**.
[b]Amino acids differ from others were highlighted with yellow background and bold.
