# Supplementary material for: Neutralizing monoclonal antibodies against the Gc fusion loop region of Crimean–Congo hemorrhagic fever virus
Source: PLoS Pathog. 2024 Feb 1;20(2):e1011948. doi: 10.1371/journal.ppat.1011948 (PMC10863865; doi:10.1371/journal.ppat.1011948)
Supplement: Attachment — Submitted filename: Response letter-2023.12.20.pdf [file ppat.1011948.s013.pdf]

Response Letter to Reviewers' Comments

(Notes: line numbers refer to revised manuscript with changes highlighted)

Reviewer's Responses to Questions

Part I – Summary

Please use this section to discuss strengths/weaknesses of study, novelty/significance, general execution and scholarship.

Reviewer #1: The manuscript by Li et al. identify several nAbs from mice, evaluate their neutralization capacity, therapeutic efficacy in mice and structurally investigate how these nAbs bind to CCHFV. Overall, the therapeutic efficacy of the identified mAbs is modest, however, authors acknowledge this. Authors mechanistic investigations of the nAb binding further highlight the epitopes on CCHFV glycoproteins that are targeted by nAbs. I would encourage authors to expand on their discussion to speculate on how they think their data predicts the therapeutic value of nAbs for CCHFV.

Reviewer #2: In this study, the authors identified two antibodies neutralizing infection by the Crimean Congo hemorrhagic fever virus, an emerging virus with a high pandemic risk. To this extent, the results shown here are of high biological significance. Having written this, the study remains highly descriptive. The results support the authors' conclusion. The experiments are, on the whole, well-controlled. The manuscript would benefit from proofreading in English style. I have a few more specific major and minor points (see below).

Response: We appreciate both reviewers very much for their careful review and valuable suggestions.

Part II – Major Issues: Key Experiments Required for Acceptance

Please use this section to detail the key new experiments or modifications of existing experiments that should be absolutely required to validate study conclusions.

Generally, there should be no more than 3 such required experiments or major modifications for a "Major Revision" recommendation. If more than 3 experiments are necessary to validate the study conclusions, then you are encouraged to recommend "Reject".

Reviewer #1: Major comments

1. Line 90-98: This section is confusing and although the methods clarify it somewhat, this results section needs to be clarified. How the mice were immunized and with what should be mentioned in the results to indicate how the hybridomas were generated.

Response: We agree with the reviewer's comments. Detailed descriptions regarding mouse immunization and generation of hybridomas have been added at the beginning of the paragraph (**lines 100-104**).

2. Line 96 – 99, what does this mean? Were hybridomas injected into mice or were antibody supernatants injected into mice? Why were mAbs generated from ascites instead of traditional in vitro production systems?

Response: We are sorry for the confusion. Hybridomas were used to immunize mice. We have clarified this in the revised manuscript (**lines 110-112).** Preparation of mAbs from ascites is a conventional method to produce mAbs with low cost and high quantities, and the method has been well established in our laboratory.

3. Line 115: How were antibodies quantified? How was purity assessed?

Response: The concentration of mAbs was determined using Bradford Protein Assay Kit (Beyotime, China) by measuring the absorbance at 595 nm using a microplate reader (BioTek Instruments). The antibody purity was assessed by coommassie brilliant blue staining with SDS-PAGE. These details have been added in the M&M section (**lines 486-489).**

4. Line 147-151: Authors show improved survival but no difference in viral loads in the liver or spleen. Although authors state that the mean was reduced by one log, 5 of 7 control treated animals had lower viral loads in the liver than the mean of Gc13 treated animals, yet 100% of control treated animals died versus only 3 of the 8 Gc13 treated animals. Is protection from death due to viral control in other tissues? Blood? Brain?

Authors may also want to highlight that they used a high dose challenge (5000 TCID50) in IFNAR-/- mice when the LD50 for CCHFV in IFNAR mice has been reported to be as low as 0.06 TCID50 (Hawman et al. 2018 Antiviral Research or Zivcec et al. 2013 JID). Although those LD50s are with other strains of CCHFV, authors may have challenged mice with ~100,000 LD50s. This may also contribute to only partial protection and also suggests that even the level of protection authors observed is notable. Authors should compare this to the challenge model used in the human nAb manuscript.

Response: We see the concern of the reviewer. The IFNAR$^{-/-}$ mice are very sensitive for CCHFV infection, with high viral replication levels in the liver and spleen (the major targeting organs), as well as in other organs/tissues. The disease onset is rapid and mice died at 4-6 dpi due to cytokine storm and multi-organ failure [1, 2]. In this experiment, unfortunately, several mice in the control group had already died for some time when collecting the tissues at the fixed time in the BSL-3

laboratory. We surmise that the low viral loads in the livers of the dead mice may be due to tissue lysis and RNA degradation by released enzymes.

We agree with the reviewer that the challenged viral dosage (5000 $TCID_{50}$) we used was high. Actually, in our infection model, 1 $TCID_{50}$ is enough to cause ~100% fatality (please see **the below Figure 1, for review only**). Therefore, we used > 500-fold LD_{99} as challenging dose this time, which may compromise the efficacy of the tested nAbs. We have compared this to the challenge models (100 PFU/IbAr10200 strain) used in the human nAb manuscript as suggested by the reviewer (**lines 375-379**).

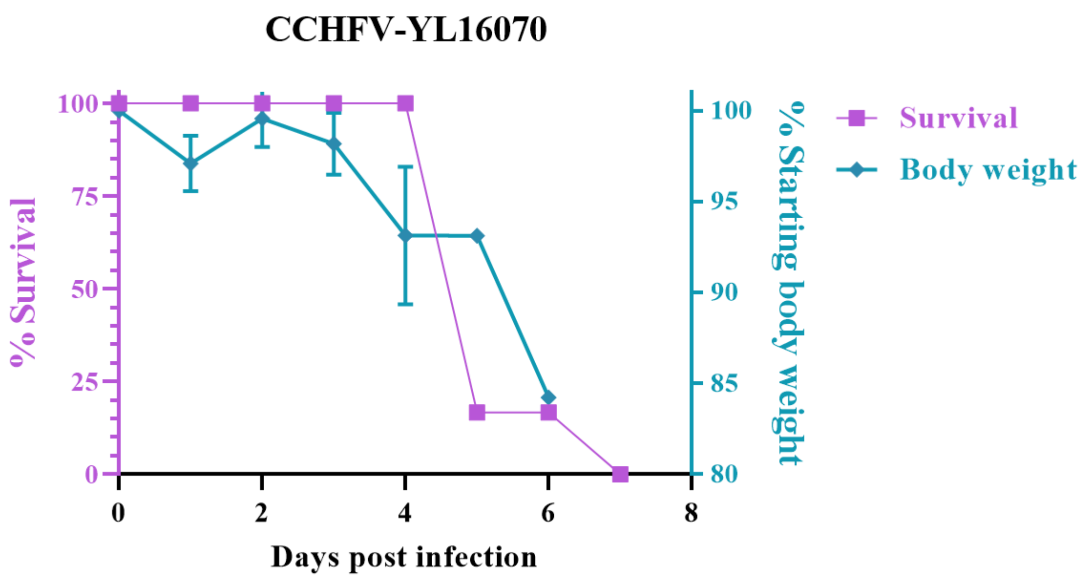

Figure 1: Survival and body weight curves of C57BL/6J-IFNAR$^{-/-}$ mice (6 mice per group) infected with 1 $TCID_{50}$ of CCHFV-YL16070 via the intraperitoneal route. The mice were monitored daily for physical condition and body weight, and were euthanized at the ethical endpoint (body weight loss reaching 20% of the starting weight).

5. Line 333-337: Could it also be due to the immunogen used in author's study? How sure are the author's that their Gc antigen was properly folded? It is possible that since the abs from convalescent survivors arose in response to authentic antigen whereas the authors abs arose from recombinant protein that the recombinant protein was improperly folded.

Response: We see the points of the reviewer. We also agree that the conformation of immunogen is a major determinant to obtain efficient mAbs. The Gc antigen we used was a trimeric ectodomain of Gc proteins expressed in eukaryotic expression system, and Cryo-EM analysis showed it was properly fold into trimers[3].Nevertheless, it is possible that there exist difference between recombinant protein and native antigen. We have added this in the **Discussion** (**lines 373-375**).

6. The discussion should wrap up with a conclusion paragraph. In the discussion I would encourage author's to speculate on how they think their data predicts the therapeutic potential of nAbs for CCHFV. Author's data adds to an overall modest efficacy of nAbs for CCHFV. Author's saw partial protection even with pre-treatment and the referenced paper using nAbs from human survivors also

saw incomplete protection as soon as mAbs were evaluated therapeutically. How does this picture compare to nAbs for other hemorrhagic fevers such as Ebola? Have authors considered screening non-neutralizing nAbs for protective efficacy?

Response: We are very grateful to the reviewer for the insightful suggestions. Triple monoclonal antibody REGN-EB3 and a single monoclonal antibody MAb114 have been approved by FDA to treat Ebola virus infection. Early administration of nAbs seems to have better clinical beneficial. Notably, both REGN-EB3 and MAb114 showed ideal therapeutic efficacy in treating the Ebola virus infection in animal models compared to the currently reported CCHFV nAbs [4-6]. Therefore, the development of more efficient CCHFV nAbs is necessary.

In addition, we also agree with the reviewer that some non-neutralizing mAbs, such as mAb targeting GP38, showed promising *in vivo* protective effect [7-9], which are also worthy to be considered for CCHF treatment, either used alone or combined with nAbs. The relevant work is underway.

Although antibody-based therapy of CCHFV has yet to be tested in clinical trials, related research is rapidly growing. Given the successful application of mAbs in the treatment of the Ebola virus, we expect promising advancement in their use in treating CCHF in future. We have added a conclusion paragraph in the final part of the Discussion (**lines 417-420; 423-425; 427-441**).

Reviewer #2:

1. In Fig. 2A, the authors assessed the viral RNA but no information is provided about the release of infectious progeny.

Response: According to the reviewer's suggestion, we determined the IC_{50} values of the three mAbs (Gc8, Gc13, and Gc35) by quantification of the release of infectious progeny using end-point dilution assay (EPDA). The results are consistent with those by quantification of viral RNA copies. We have added the figure as the new **Figure 2B** and described it in the **Results** (**lines 139-141**) and **Methods** section (**lines 521-524**).

2. Fig. 2B without any quantification is not informative. It's hard to see any difference by eye in these series of fluorescence microscopy images.

Response: We agree with the reviewer. We have replaced it by IC_{50} determination using viral titration method (the new **Figure 2B**).

3. Fig. 3 missed non-infected mice, which would make it possible to assess the effect of the antibodies on the survival of animals without the virus.

Response: We see the concern of the reviewer. We have evaluated the effect of the three mAbs (50 mg/kg, the same dose as in the experiments with virus challenge) on the survival of animals in the absence of CCHFV infection. Daily monitor of physical condition and body weight change of the mice showed **no difference** between antibody group and the PBS group. Thus, the three mAbs seem

to be safe to the animals (please see **S1 Figure**). We have added this data in the manuscript as **S1 Fig** (**lines 158-160**).

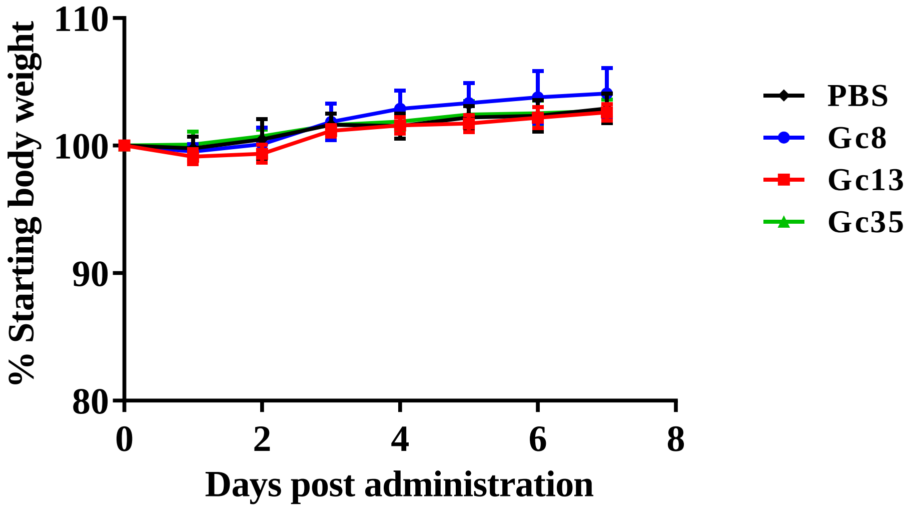

+

S1 Figure: Body weight curve of C57BL/6J-IFNAR$^{-/-}$ mice (5 mice per group) administered with Gc8, Gc13 and Gc35 at a dose of 50 mg/kg, and the same volume of PBS control, via the intraperitoneal route.

4. Similar to Point 2, Fig. 4B is not very informative as it is.

Response: The purpose of Fig. 4B (IFA) is to show that the mAbs can recognize native Gc proteins in CCHFV infected cells. Although it is not a quantitative experiment, we believe the result provides some useful information and also showed the specificity of the antibodies.

5. Fig. 6 is not convincing, essentially because it is not possible to see syncytia on IF pictures shown in 6C.

Response: We see the concern of the reviewer. We are sorry that the resolution of the previous IF pictures are not high enough. To confirm the result, we have repeated this assay, outlined each syncytium in 10 randomly-chosen fields (as represented in **the new Figure 6C**), and re-calculated the data (the new **Figure 6B**). The conclusion is similar with the previous result. We hope the current data can satisfy the reviewer.

6. The TEM work shown in Figures 7 and 8 is too succinctly described. In general, the Results section lacks information and description in many places.

Response: We have supplemented more detailed descriptions in the **Results** section (**lines 317-323**), as well as in the **M&M** section (**lines 654-658**). In addition, a **new S6 Table** on residues/atoms involved in hydrogen bonds between Fab and the fusion loops has been added and discussed.

7. The discussion is overall a retelling of the results.

Response: We appreciate the reviewer for the valuable suggestion. The Discussion section has been improved by minimizing repetition of the results and expounding on result discussion (**lines 334-337, 344-351, 373-390, 398-401, 417-441**). Particularly, we have added a concluding paragraph summarizing the state of the art of mAbs in treating Ebola virus and predicting the therapeutic potential of mAbs for CCHFV (please see the **Response #6 to Reviewer 1**).

Part III – Minor Issues: Editorial and Data Presentation Modifications
Please use this section for editorial suggestions as well as relatively minor modifications of existing data that would enhance clarity.

Reviewer #1:

1. Abstract is too detailed, I would consider shortening it.

Response: Thanks for the suggestion. The abstract has been simplified to be more general.

2. Line 56: clarify that it is the Nairoviridae family in the Bunyavirales order.

Response: It has been revised according to the reviewer's suggestion (**line 63**).

3. In figure 7 would recommend highlighting the fusion loop to make it clearer to the reader when author's state the abs target the fusion loop. Particularly panel C?

Response: According to the reviewer's suggestion. We have revised the **Fig. 7C** by adding an inset of the three fusion loops from the green sGc molecule.

4. Line 306: sulking should be suckling

Response: Thanks for the kind reminder. It has been corrected (**line 339**).

Reviewer #2:

1. Line 34 (abstract), "confirmed" is inappropriate. "Showed", "indicated" etc. would be better.

Response: It has been revised according to the reviewer's suggestion (**line 39**).

2. The Author Summary is too specialized and not accessible enough to a wide audience.

Response: The Author Summary part has been improved to be more readable.

3. The Results section should begin with a brief explanation of the origin of hybridomas. This is done, but far too late in the discussion.

Response: As also suggested by Reviewer 1 (**Response #1**), we have added more detailed descriptions of generation of mAbs at the beginning of the Results section (**lines 100-104, 111**).

4. In the context of work on Gc-neutralizing antibodies, the first published structure for CCHFV Gc should be cited (PMID: 34793197).

Response: We agree with the reviewer. This is an important reference, which was already cited in Introduction and Discussion part as **Reference No. 12** (**lines 89 and 406**).

5. The term "strains" to describe antibodies seems inappropriate to me.

Response: We agree with the reviewer. The term "strains" has been replaced by "mAb" (**lines 110 and 123**).

6. In Fig. 5A, the term "naked" to describe the antibodies that are not biotinylated also seems inappropriate to me.

Response: We agree with the reviewer. The term "naked mAb" was replaced by "unlabeled mAb" throughout the **text** (**lines 223 and 576**) and **Figure 5A**.

References:

1. Zivcec M, Safronetz D, Scott D, Robertson S, Ebihara H, Feldmann H. Lethal Crimean-Congo Hemorrhagic Fever Virus Infection in Interferon α/β Receptor Knockout Mice Is Associated With High Viral Loads, Proinflammatory Responses, and Coagulopathy. J Infect Dis. 2013;207:1909-1921.
2. Wang Q, Cao R, Li L, Liu J, Yang J, Li W, et al. In vitro and in vivo efficacy of a novel nucleoside analog H44 against Crimean–Congo hemorrhagic fever virus. Antiviral Res. 2022;199:105273.
3. Li N, Rao G, Li Z, Yin J, Chong T, Tian K, et al. Cryo-EM structure of glycoprotein C from Crimean-Congo hemorrhagic fever virus. Virol Sin. 2022;37:127-137.
4. Corti D, Misasi J, Mulangu S, Stanley DA, Kanekiyo M, Wollen S, et al. Protective monotherapy against lethal Ebola virus infection by a potently neutralizing antibody. Science. 2016;351:1339-1342.
5. Pascal KE, Dudgeon D, Trefry JC, Anantpadma M, Sakurai Y, Murin CD, et al. Development of Clinical-Stage Human Monoclonal Antibodies That Treat Advanced Ebola Virus Disease in Nonhuman Primates. J Infect Dis. 2018;218:S612-S626.
6. Fels JM, Maurer DP, Herbert AS, Wirchnianski AS, Vergnolle O, Cross RW, et al. Protective neutralizing antibodies from human survivors of Crimean-Congo hemorrhagic fever. Cell. 2021;184:3486-3501.
7. Golden JW, Shoemaker CJ, Lindquist ME, Zeng X, Daye SP, Williams JA, et al. GP38-targeting monoclonal antibodies protect adult mice against lethal Crimean-Congo hemorrhagic fever virus infection. Sci Adv. 2019;5:eaaw9535.

8. Mishra AK, Moyer CL, Abelson DM, Deer DJ, El Omari K, Duman R, et al. Structure and Characterization of Crimean-Congo Hemorrhagic Fever Virus GP38. J Virol. 2020;94:e02005.
9. Durie IA, Tehrani ZR, Karaaslan E, Sorvillo TE, McGuire J, Golden JW, et al. Structural characterization of protective non-neutralizing antibodies targeting Crimean-Congo hemorrhagic fever virus. Nat Commun. 2022;13:7298.
